# Correlative imaging reveals metal dyshomeostasis and altered zinc coordination environments in a pre-clinical Type 2 diabetes model

**DOI:** 10.1093/mtomcs/mfaf043

**Published:** 2026-01-02

**Authors:** Gaewyn Ellison, Arazu Sharif, Meg Willans, Ashley Hollings, Ryu Takechi, Keith Bambery, Valerie Mitchell, Daryl L Howard, Mark J Hackett

**Affiliations:** School of Molecular and Life Sciences, Faculty of Science and Engineering, Curtin University, Bentley, WA, Australia; Curtin Medical Research Institute, Curtin University, Bentley, WA, Australia; Curtin Medical Research Institute, Curtin University, Bentley, WA, Australia; School of Population Health, Faculty of Health Sciences, Curtin University, Bentley, WA, Australia; School of Molecular and Life Sciences, Faculty of Science and Engineering, Curtin University, Bentley, WA, Australia; Curtin Medical Research Institute, Curtin University, Bentley, WA, Australia; School of Molecular and Life Sciences, Faculty of Science and Engineering, Curtin University, Bentley, WA, Australia; Curtin Medical Research Institute, Curtin University, Bentley, WA, Australia; Curtin Medical Research Institute, Curtin University, Bentley, WA, Australia; School of Population Health, Faculty of Health Sciences, Curtin University, Bentley, WA, Australia; Australian Synchrotron, ANSTO, 800 Blackburn Road, Clayton, VIC 3168, Australia; Australian Synchrotron, ANSTO, 800 Blackburn Road, Clayton, VIC 3168, Australia; Australian Synchrotron, ANSTO, 800 Blackburn Road, Clayton, VIC 3168, Australia; School of Molecular and Life Sciences, Faculty of Science and Engineering, Curtin University, Bentley, WA, Australia; Curtin Medical Research Institute, Curtin University, Bentley, WA, Australia

## Abstract

Zinc ions are highly abundant in pancreatic islet tissue, and multiple lines of evidence link loss of zinc homeostasis to poor glucose regulation in both type 1 and type 2 diabetes. Two major islet zinc-binding proteins, insulin and metallothionein, play crucial roles in beta cell function and glucose regulation. Here we used X-ray fluorescence microscopy (XFM) to map zinc and five additional elements (Cl, K, Ca, Fe, and Cu) to compare the metallome of exocrine, peri-islet and islet regions in young and old, non-diabetic control and diabetic (*db/db*) mice. We also determined the main forms of zinc found in pancreatic tissue using X-ray absorption near-edge structure (XANES) spectroscopic imaging. This allowed investigation of the relationship between zinc speciation and its protein ligands using correlative immunofluorescent imaging to assess whether zinc coordination may play a role in diabetes pathology. The anticipated depletion of zinc in young diabetic islets was accompanied by a significant decrease in insulin expression and increase in metallothionein expression. A parallel change in the contribution of cysteine vs histidine zinc speciation was also observed. Counter-intuitively, zinc abundance and speciation appeared to normalise in old diabetic animals with more advanced disease, despite large differences in labile zinc-binding protein content. These results are consistent with disrupted zinc coordination, where metallothionein-regulated muffling to minimise ionic activity is overwhelmed and zinc binds to unidentified ligands in histidine-like conformations. This opens future study questions focussed on the complex interplay between labile zinc, metallothionein, and oxidative mechanisms that may interfere with normal zinc homeostasis.

## Graphical Abstract

**Graphical Abstract:**
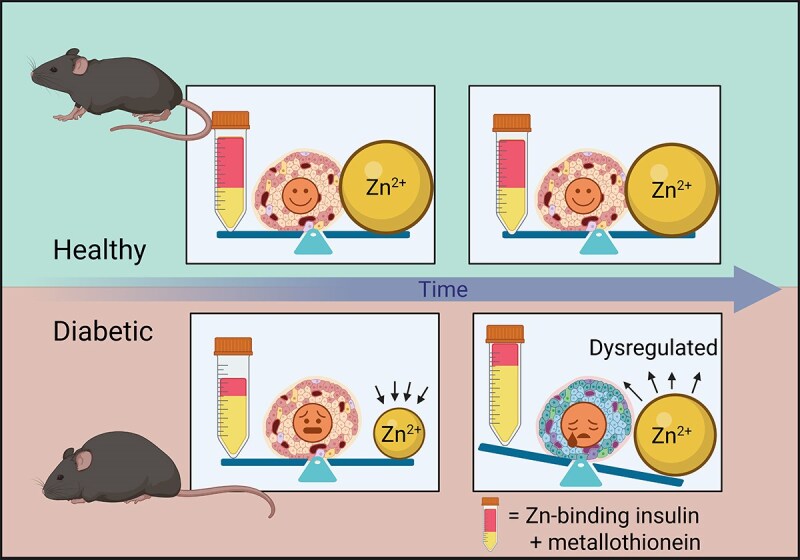
Created in https://BioRender.com

## Abbreviations

MT: metallothionein
XANES: X-ray absorption near-edge structure
XFM: X-ray fluorescence microscopy
Zn^2+^: inorganic zinc ions
ZnT8: a zinc efflux transporter important to zinc accumulation in beta cell granules also known as solute carrier family 30 member 8 (SLC30a8)
T2DM: Type 2 diabetes mellitus

## Introduction

Zinc (Zn) is a key biometal, with catalytic, co-catalytic or structural roles [1] in around 3 000 human proteins [2]. Among its many roles is its involvement in glucose regulation. Pancreatic islets, and more specifically beta cells, contain among the highest zinc concentrations found in the human body, measured at millimolar levels [3]. Zinc is required for the biosynthesis and efficient storage of insulin within insulin granules in most species [4]. The immature form of insulin, known as proinsulin, has the capacity to bind 30 zinc ions [4], and once converted to mature insulin, is stored in acidic granules [5] in crystalline form as a hexamer complexed with two zinc ions [4,6]. Each beta cell has around 13 000 granules [7] filled with crystalline insulin bound to Zn in a 3:1 ratio [4]. When this insulin is secreted, an increase in pH in the extracellular environment facilitates the dissociation of insulin from zinc to its active, soluble monomer form [5].

Secreted zinc has autocrine and paracrine influences in islets, with a role in the regulation of insulin and glucagon secretion [6,8–10], although the exact nature of its signalling activities is still under debate [11,12]. Secreted Zn has also been shown to have insulin-mimetic [13] and hepatic regulatory [14] effects, suggesting a range of signalling activities that are not well understood. It is likely that any endocrine effect of secreted Zn would be by means of specific Zn complexes rather than by concentration alone [15].

Zn accumulates in beta cell insulin granules by means of a specific Zn transporter known as solute carrier family 30 member 8 (SLC30a8), or ZnT8 [16], also expressed in alpha cells [17]. A beta cell-specific ZnT8 knock-out/overexpression study remarkably found that Zn secretion was more relevant to glucose tolerance than insulin secretion, possibly due to its influence on hepatic insulin clearance [18]. Additional extra-granular pools of metabolically labile Zn^2+^ also occur in islets [19]. These Zn ions are buffered or ‘muffled’ by binding with variable affinity to metallothioneins, which are exquisitely tuned to regulate Zn^2+^ over a wide range of concentrations [20]. Roles fulfilled by the transient pool of labile Zn^2+^ include signalling, use as enzyme co-factors, and, in beta cells, insulin crystallisation and storage.

Zn dysregulation has been associated with diabetes. Genome wide association studies have linked mutations in ZnT8 to increased or decreased risk of Type 2 diabetes mellitus (T2DM) [21–24]. Mouse knockout studies have confirmed this link [25]. In addition, autoantibodies to ZnT8 were found in 60-80% of paediatric patients with type 1 diabetes, compared to 2% of healthy controls [26], and the presence of these autoantibodies can be used both for screening and as a prognostic marker in high risk populations [27].

Zn supplementation has been studied for its potential use in the treatment of diabetes [28–30] due to the association of low plasma Zn with diabetes [31,32] and diabetic complications such as polyneuropathy [33]. Zn deficiency is thought to be partly due to urinary loss of Zn during hyperglycaemia [6,34]. Zn supplementation reduced the risk of diabetes in women [35] and after streptozotocin injection in mice [36] and has been known to improve glycaemic control in diabetic mice [30,37] and humans (reviewed in [38]). However, not all studies have shown beneficial effects of Zn supplementation [39–41], and its efficacy to treat metabolic conditions is still debated [42].

Despite the many studies of Zn in diabetes and in pancreatic islets, until recently it has been difficult to directly measure or map Zn in islets. This is due in part to the labile nature of the ‘free’ Zn pool, which is at risk of being washed away in protocols using dyes or stains, and also to limitations of the stains available, which may reveal only a restricted portion of the Zn pool [43,44]. Improvements in X-ray optics and detector electronics has meant that the spatial resolution of X-ray fluorescence microscopy (XFM) has improved to the point where cellular and sub-cellular mapping of trace elements is now possible within an experimentally realistic time frame [45]. Further, when coupled with X-ray absorption near-edge structure (XANES) spectroscopy, the coordination environment of metal ions can be studied [46,47].

In biological systems, Zn is only present with a valence state of + 2 and is redox-inert due to its filled d-shell orbital [1,48], although it still influences redox biochemistry [49]. Zn speciation reflects function and can affect its bioavailability and action [28]. For example, catalytic zinc is usually bound to histidine residues whereas structural Zn is associated almost exclusively with cysteine residues [50]. Speciation can be determined by spectral data collected using Zn-specific XANES imaging, since spectra are influenced by the oxidation status and geometry of the species of Zn within the X-ray’s influence [46,51,52].

The spectra of a given ROI represent an average of all the Zn variations within that region. It is possible to deconvolute speciation and estimate the main Zn ligands contributing to the spectrum by fitting the spectrum from a specific ROI to a selection of XANES spectra generated from model solutions of biologically relevant Zn compounds predicted to be found in the sample. These standard solutions represent chemical equilibria, since Zn ligands are added in excess and several binding variations are usually possible. While this does not conclusively inform regarding the number of coordinating molecules, the spectra predictably represent Zn speciation.

While the focus of this study was on Zn, other metal ions have also been associated with increased risk of diabetes. For example, iron (Fe) influences glucose metabolism, such that increased Fe stores are associated negatively with insulin resistance and positively with risk of T2DM [53–55]. Copper (Cu) is another crucial trace element [56] which requires sensitive regulation to manage oxidative stress. A competitive relationship exists between copper and zinc ions due to their similar properties [57]. Zinc toxicity from excessive dietary intake manifests as copper deficiency [58], and excessive copper accumulation (such as in copper toxicosis in sheep or Wilson’s disease in humans) can be treated by zinc supplements [57]. This demonstrates the benefit of studying multiple trace elements concurrently.

The aim of this study was to characterise elemental homeostasis within islets from young or old, non-diabetic control or diabetic mice using XFM. Further, to identify the major forms of Zn in islets using XANES spectroscopy to see whether Zn speciation changes were associated with type 2 diabetes. Changes in Zn speciation can be associated with pathophysiological consequences, and much is still to be discovered about biological forms of Zn, particularly in the labile pool [48]. Increased understanding of the role of Zn in diabetes may provide new therapeutic targets for this condition, which is escalating globally [59], and for which there is still no cure.

## Methods

### Animals

Pancreatic tissue was collected from male *db/db* (Db) and *db/+* (Het) mice obtained from Jackson Laboratory and bred at the Animal Resources Centre (WA). This is a well-established type 2 diabetes mouse model on a C57Bl/6 J background [60]. These mice contain a mutation in the receptor for leptin, a satiation hormone [61]. The mice are hyperphagic and become obese, displaying clinical symptoms of pre-diabetes from as early as 2-4 weeks of age [62]. Mice were enrolled in the study at 5 weeks of age, housed in a ventilated vivarium with a 12-hour day/night cycle and maintained on standard chow with *ad libitum* access to food and water until 14 (young) or 28 (old) weeks of age. They were humanely killed by cervical dislocation under isoflurane anaesthesia without fasting. All animal work was conducted according to the Australian code for the care and use of animals for scientific purposes, 8th edition [63]. The use of animals was approved by Curtin Ethics Committee (Ethics approval number ARE2020-12).

### Tissue collection and sample preparation

Pancreatic tissue was collected rapidly, snap-frozen in liquid nitrogen-cooled isopentane and stored at -80 °C until needed. A series of adjacent tissue slices 10 μm thick were cut on a cryo-microtome at -16 °C and briefly melted onto glass slides or silicon nitride windows (5×5 mm x 1 μm thick membrane, Melbourne Centre for Nanofabrication) for immuno-fluorescent staining or XFM and XANES spectroscopy, respectively. To avoid loss or altered distribution or coordination of elements [64–66], no perfusion, buffer or saline washing, or chemical fixation step was used. Silicon nitride windows were stored with desiccant at -80 °C until being shipped frozen to the ANSTO Australian Synchrotron campus at Clayton, Victoria. Glass slides were stored at -80 °C until needed.

### Elemental mapping, chemically specific XFM and XANES spectroscopy at the ANSTO-Australian Synchrotron

Elemental mapping, chemically specific XFM and XANES mapping was performed at the X-ray fluorescence microscopy beamline at the ANSTO-Australian Synchrotron [67] using similar methods to those optimised for imaging labile transition metals in brain slices [68]. Unfixed tissue samples were kept frozen under a nitrogen vapour cryostream (max temp, -40 °C) during scanning. Elemental mapping XFM was conducted above the Zn K-edge at 9803 eV with a dwell time of 2 ms and a 2 μm step size. The X-ray beam was focussed using Kirkpatrick—Baez (KB) mirrors, achieving a focussed spot size estimated to be 2 µm at 2σ. Fluorescence (K_α_ emission) was detected using a Vortex-EM detector in 45-degree geometry. Elemental fluorescence maps were converted to quantitative elemental maps in GeoPIXE (CSIRO, Australia) using Fe and Mn standard foils (Micromatter, Canada), which were analysed under the same conditions and geometry as tissue samples. The conversion algorithm accounted for the composition and density of the silicon nitride substrate and the thickness and approximate composition of the tissue sample (C22H10N2O4).

Following XFM elemental mapping of pancreas tissue sections, which revealed location of islets, a sub-region of interest was then selected for chemically specific XFM and XANES spectroscopic analysis, collecting data from 98 scans across the Zn K-edge between 9630-9803 eV (0.5 eV steps across the Zn K-edge and dwell time and step size as for XFM). Data was co-collected in transmission mode from an elemental Zn foil placed downstream of the tissue samples to use as a reference for energy calibration for XANES spectra.

Using GeoPixe (http://nmp.csiro.au/GeoPIXE.html), quantitative data was extracted as TIFF files of per-pixel elemental areal density in ng.cm^-2^ to use for further analysis as previously described [69]. Fluorescence data within regions of interest (ROI) was normalised to Compton (inelastic) scatter to account for variations in section thickness. The process for normalisation to Compton scatter was as follows: within each ROI, elemental fluorescence values were divided by Compton scatter values for the same ROI, then multiplied by the group average Compton scatter for the relevant ROI (to provide values similar in magnitude to the original concentration). Compton scatter had a mean coefficient of variation (CV%) of 12% across all ROI’s. ImageJ [70] was used to extract fluorescence intensity data and generate XANES spectra for the regions of interest. Fitting of Zn spectra to a library of biologically relevant Zn model solutions was performed using EXAFSPAK (https://www-ssrl.slac.stanford.edu/~george/exafspak/exafs.htm), as described previously [46]. Similar to our previous results when examining Zn speciation in brain tissue sections [46], and also similar to other biological samples [71], the Zn K-edge XANES spectra could be adequately fitted to several main components. Extensive combinations of fits were trialled, using our library of standard solutions and model compounds (Table S1), however the fits with lowest residuals were obtained from fitting two major components that reflect Zn histidine coordination and Zn cysteine coordination. Principal component analysis (PCA) was undertaken to enable chemically specific spectral maps to be generated via single value decomposition using MANTiS version 2.3.02, as described [72,73].

### *Collection of bulk XANES spectra* from *standard solutions*

A stock solution of zinc nitrate (1 mM) was prepared from Zn(NO_3_)_2_.6H_2_O (Sigma) and used as the source of Zn^2+^ for the preparation of standard solutions, as described previously. The preparation and analysis of standard solutions was undertaken as part of a broader research program analysing Zn^2+^ speciation in pancreas, brain [46], and plant tissues. Of relevance to this study were standard solutions prepared with Zn^2+^ in the presence of excess histidine and excess cysteine, however standard solutions modelling Zn^2+^ coordinated to Cl^−^, phosphate, glutamate, citrate, and water (pH 7 and pH 13) were also examined (Supplementary Table S1). The additional standard solutions were prepared such that the ligands had a concentration of approximately 10 mM (i.e. ligand to metal ratio of 10:1). We have used the terminology Zn(histidine) and Zn(cysteine) to represent all forms of Zn that would exist in the presence of an excess of histidine (Zn(histidine)) or cysteine (Zn(cysteine)).

All Zn K-edge XANES spectra were collected at the Australian Synchrotron, using the X-ray Absorption Spectroscopy beamline equipped with a 1.9T wiggler and Si(111) double crystal monochromator (DCM), as previously described [46]. Specifically, the standard solutions were mounted at 45° to the incident X-ray beam, in a liquid Helium-cooled cryostat (measurements were recorded at ∼12-15 K). Kα fluorescence emission from the standard solutions was recorded using a Canberra liquid nitrogen-cooled 100-pixel monolithic solid state Ge detector. X-ray energy calibration was performed against the absorption spectrum of Zn metallic foil, calibrated to first inflection peak at 9660.7 eV. The XANES spectra were recorded across the K-edge white line features from 9650—9700 eV at 0.3 eV steps (500 ms per data point). Spectra were base-line subtracted and normalised to a post-edge jump of 1, using EXAFSPAK, as previously described [74].

### Immunohistochemistry

Sections (10 μm) on glass slides were fixed for 10 min in 4% paraformaldehyde (PFA). PFA was removed by washing in Tris-buffered saline containing 0.2% Tween-20 (TBST), and non-specific binding was blocked for 1 h using 10% goat serum and 1% bovine serum albumin (BSA) in TBST. Primary antibodies were applied at pre-optimised concentrations (Supplementary Table S2) and incubated at 4 °C overnight. Samples were then washed and incubated with secondary antibodies for 2 h at RT. Samples were mounted using ProLong™ Gold Antifade Mountant with DAPI (Life Technologies). Imaging was performed using a ZEISS Axio Scan.Z1 instrument at the Curtin MRI Microscopy and Histology facility. ZEN version 3.2 and ImageJ [70] were used for image analysis.

Post-XFM staining for insulin and glucagon was also performed on the same samples mounted on silicon nitride windows to confirm islet regions. Samples were allowed to air dry after XFM and XANES scanning and were stored at ambient temperatures in the dark for 2-4 weeks prior to staining according to the protocol above.

### Plasma glucagon, insulin and glucose measurement

Blood was collected in EDTA-coated syringes by cardiac puncture under deep isoflurane anaesthesia and centrifuged at 1200 x *g* for 10 minutes at 4 °C. The plasma was then collected and stored at -80 °C until assayed. Glucagon and insulin ELISA kits (Mercodia, from Sapphire Bioscience, Australia, catalogue # 10-1281-01 and 10-1247-01, respectively) were used according to manufacturer’s instructions to measure plasma glucagon and insulin. Blood glucose was assayed using an enzymatic protocol (Cayman Chemical Glucose Colorimetric Assay Kit; Catalogue # 10 009 582).

### Statistical analysis

ImageJ was used for image analysis. Quantitative data are reported as mean ± SEM and statistical analysis was performed using 2-tailed Student’s t tests or 2-way ANOVA followed by Tukey’s multiple comparisons test in GraphPad Prism version 9.2.0 for Windows, San Diego, California, USA. A nominal *p*-value of 0.05 was used to designate significance.

## Results

### Post-XFM insulin staining

**Figure 1:**
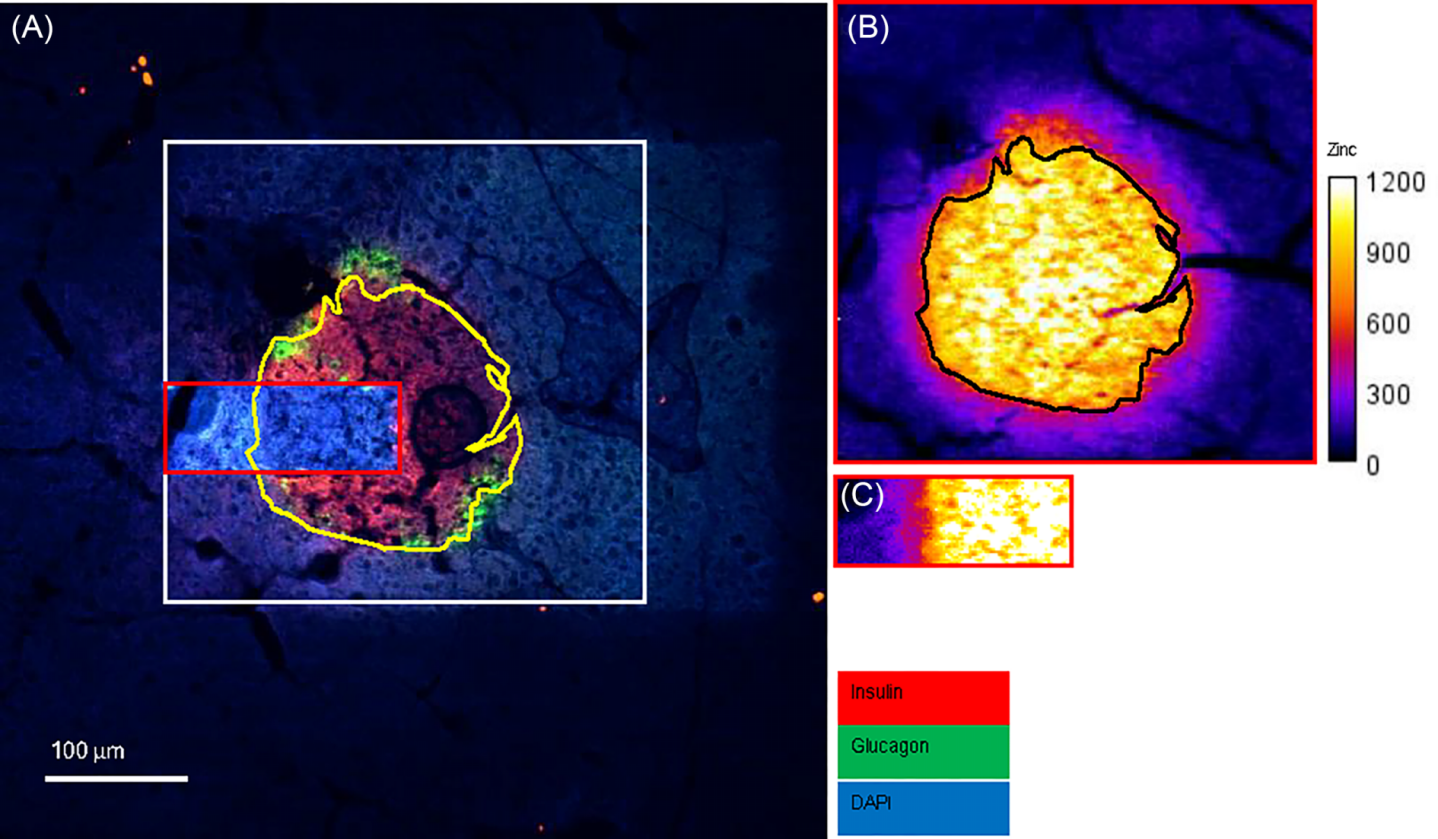
**Insulin staining co-localised with high zn regions in XFM maps**. Tissue sections were air-dried after XFM and XANES imaging and were stained (**A**) with anti-insulin (red) and anti-glucagon (green) antibodies and DAPI (blue) counterstain to reveal cell nuclei. Insulin staining correlated very closely with areas of high Zn staining (**A**, yellow line and **B** black line surrounding islet), despite increased fluorescence associated with X-ray damage (large box, white in **A** and red in **B**) and shrinkage and cracking associated with air-drying. The area used for XANES imaging (**C**), and small red rectangle in (**A**) received ∼100-fold more exposure to X-rays, and was not compatible with subsequent immunofluorescence staining. scale bar = 100 μm and applies to all images.

Regions of interest for XFM and XANES spectroscopic maps were selected according to Zn fluorescence (K_α_ emission) and expected islet morphology and location based on prior insulin staining on adjacent tissue slices. Post-XFM, the same tissue samples used for XFM were stained for insulin and glucagon using immunofluorescence, and images were overlaid on Zn^2+^ maps collected by XFM to confirm that the regions selected co-localised with insulin staining (Fig 1). There was a high degree of correlation between the regions of high Zn signal and insulin staining.

### Cl, K, Ca, Fe, Cu and Zn elemental mapping

**Figure 2:**
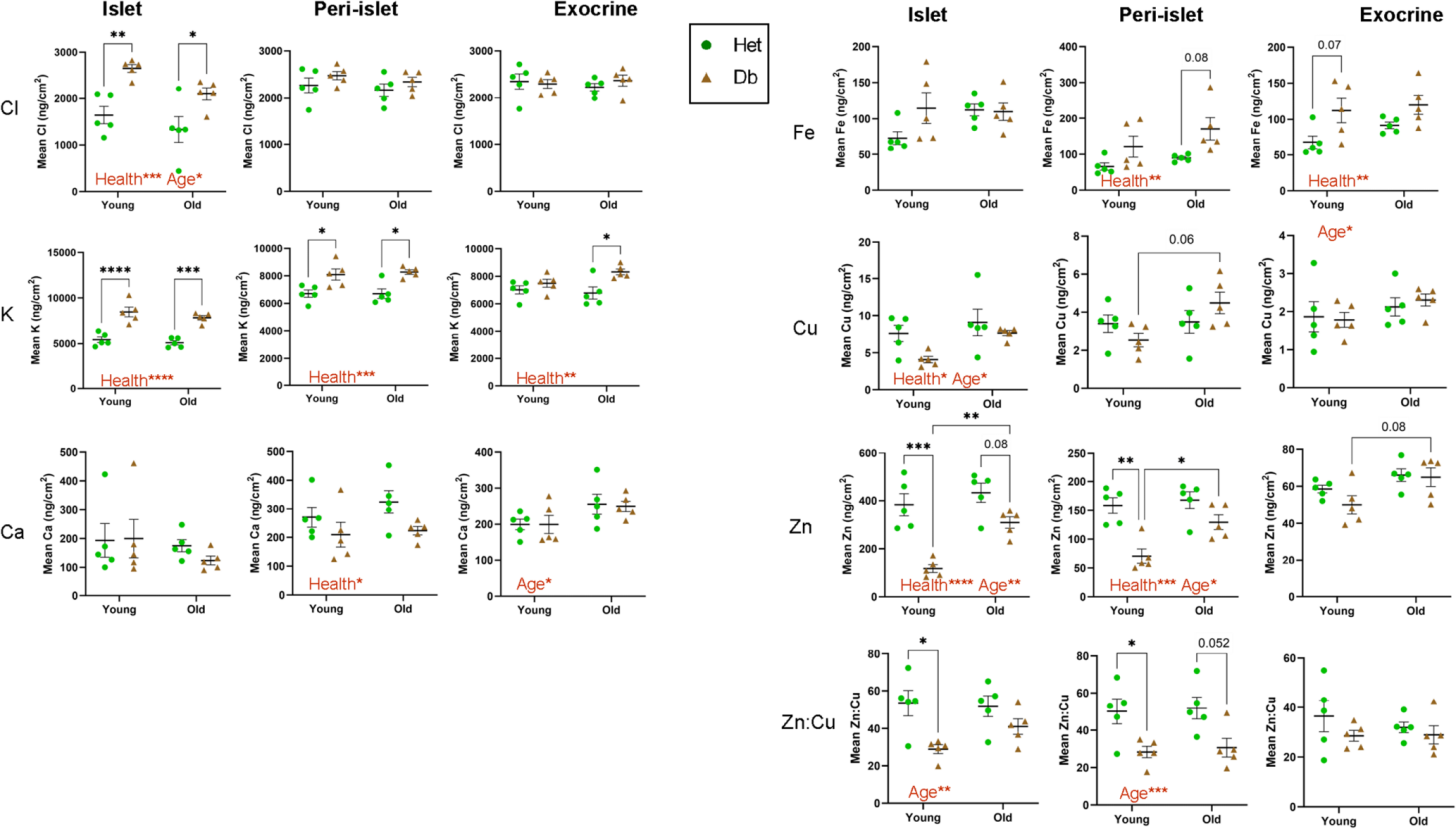
**Quantitation of 6 elements (Cl, K, Ca, Fe, Cu and Zn) in the healthy or obese, young or aged endocrine and exocrine murine pancreas**. Data was normalised to Compton (inelastic) scatter to account for any variation in section thickness. Significant differences between control (Het) and diabetic (Db) or young and old groups collectively are labelled ‘Health’ or ‘Age’, respectively, followed by an indication of significance. Het: control mice (heterozygous), green circles; Db: obese, diabetic (db/db mouse model for Type 2 diabetes), tan triangles. * *P* < 0.05, ** *P *< 0.01, *** *P* < 0.001, **** *P *< 0.0001.

**Figure 3:**
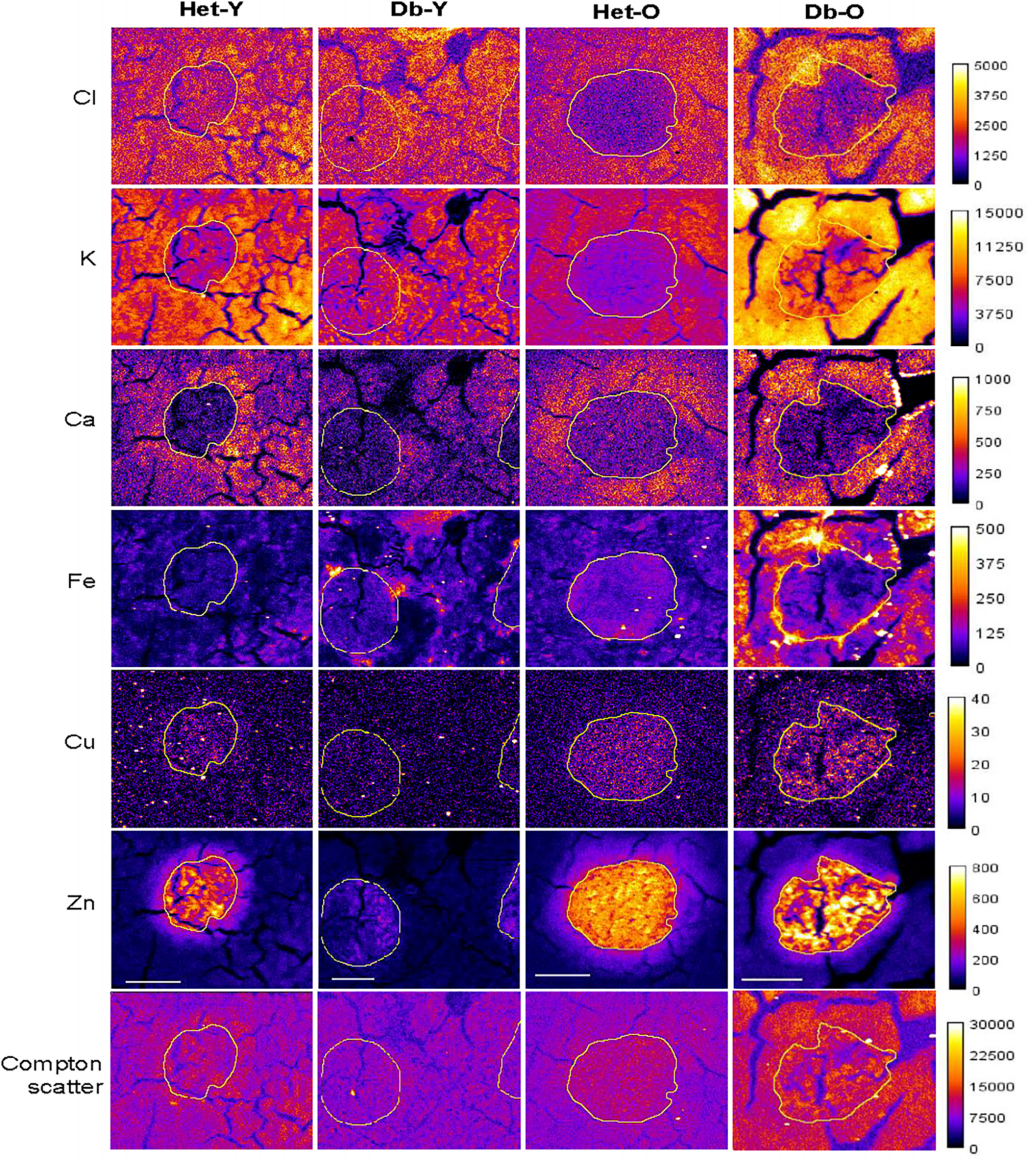
**Elemental maps of 6 elements (Cl, K, Ca, Fe, Cu and Zn)**. Representative images from each group demonstrate the distribution of 6 elements in the endocrine and exocrine regions of murine pancreas from healthy or obese, young or old animals. Islet areas are indicated, and Compton (inelastic) scatter is included for comparison. Within each element, all colour scales are relative. Scale bars in the Zn maps apply to each element and represent 100 μm. Calibration bars represent grey values and all images use ‘Fire’ look-up tables.

All six elements (Cl, K, Ca, Fe, Cu, Zn) were analysed within three regions of interest (ROIs) in five animals per group (Figs. 2 and 3). These ROIs were selected according to Zn fluorescence intensity, representing the islet (high Zn), the peri-islet (medium Zn) and exocrine regions (low Zn). Supplementary Table S3 contains a summary of elemental mapping data in ng.cm^−2^ given as mean ± SEM.

### Chloride

There was a significant increase in Cl within islets of Db compared to Het mice (1.6-fold, *p* < 0.001), such that both the young (*p* < 0.01) and old (*p* < 0.05) Db groups had significantly increased Cl compared to their non-diabetic, age-equivalent litter mates. There were no changes in Cl in the peri-islet or exocrine regions. Exocrine regions from non-diabetic mice had significantly elevated Cl compared to islet regions (1.5-fold, *p* < 0.001), but this difference was not present in Db pancreatic tissue.

### Potassium

Potassium (K) was significantly elevated in the Db compared to the Het islets overall (1.6-fold, *p* < 0.0001), this being significant in both age groups within the islet ROI (1.6-fold, *p* < 0.0001 and 1.6-fold, *p* < 0.001 for young and old, respectively). This elevation of K in the Db mice persisted within the peri-islet region (1.2-fold, *p* < 0.05 in both young and old) and, in the old groups only, also in the exocrine region (1.2-fold, *p* < 0.05). K was 1.3-fold higher (*p* < 0.001) in exocrine than endocrine tissue in non-diabetic control mice but the contrast in K concentration between these regions was lost in Db mice because the increase in K concentration in islets surpassed that of the exocrine regions.

### Calcium

Calcium (Ca) content in islets was not different between individual groups, however, there was a 1.4-fold higher Ca content in non-diabetic compared to Db groups in the peri-islet region (*P* < 0.05). In the exocrine region, old animals had a 1.3-fold elevation in Ca compared to young animals (*p* < 0.05). Interestingly, Ca was significantly higher in the peri-islet than the islet regions. There was an average 1.9-fold (*P* < 0.0001) or 1.7-fold (*P* < 0.01) increase in Ca concentration in the peri-islet region compared to the islet itself in non-diabetic control and diabetic mice, respectively.

### Iron

Iron (Fe) was elevated in peri-islet and exocrine regions of Db compared to Het animals (1.9-fold, *P* < 0.01 and 1.5-fold, *P* < 0.01, respectively), though individual age-group differences did not reach statistical significance. Distribution was highly irregular and may be influenced by the presence of blood vessels.

### Copper

Copper (Cu) was found in higher concentrations in the islets than exocrine or peri-islet regions in both non-diabetic and Db groups, such that there was a gradient of concentration from the islet (highest concentration) towards the exocrine region (lowest concentration). This resulted in a 4-fold (*P* < 0.0001) and 2.9-fold (*P* < 0.001) difference in Het and Db groups (respectively) between the islet and exocrine regions. Cu was also more abundant in non-diabetic control compared to diabetic islets (1.4-fold, *P* < 0.05), though this did not reach statistical significance in either age-group. Similarly, there was an age-dependent increase in islet Cu content (1.4-fold, *P* < 0.05), though this did not reach statistical significance in either health group. Diabetic peri-islet regions tended towards decreased Cu in young animals and increased Cu in old animals compared to their non-diabetic, age-matched controls, resulting in a 1.8-fold difference between them that approached significance (*P* = 0.06).

### Zinc

Similarly to Cu (but with concentrations two orders of magnitude higher), Zn was present in islets in high concentrations and displayed a concentration gradient reducing through peri-islet towards exocrine regions, where it is thought to play a role in inhibiting proteases stored within vesicles in acinar cells [48]. As expected, islet regions of non-diabetic control animals had greatly increased Zn content (383 ± 46 and 433 ± 40 ng/cm^2^ for young and old, respectively), representing a 6.6-fold increase compared to exocrine regions (*P* < 0.0001). Diabetic islet regions also demonstrated a significantly elevated Zn content in islet compared to exocrine regions (118 ± 16 and 310 ± 24 ng/cm^2^ for young and old, respectively), representing a 2.4-fold (*P *< 0.5) and 4.8-fold (*P *< 0.0001) elevation in Zn content compared to exocrine regions, respectively. Islets from the young non-diabetic control animals had significantly more Zn compared to their Db counterparts (3-fold, *P *= 0.0002). Interestingly, the difference was somewhat ameliorated in the old diabetic animals, such that a statistical difference was found between old and young diabetic islets (2.6-fold, *P *< 0.01) but not between islets in the two old groups (non-diabetic control and diabetic, *P *= 0.08). A similar pattern emerged in the peri-islet region, where non-diabetic control animals again had significantly more Zn overall (1.6-fold, *P *< 0.001), but only young diabetic tissue was significantly Zn depleted relative to non-diabetic young and old controls and old Db mice (*P *< 0.01, *P *< 0.001 and *P *< 0.05, respectively). There was no significant difference in the Zn content of exocrine regions between the four groups, although old mice demonstrated an overall 1.2-fold increase compared to young animals (*P *< 0.05).

### Zn: Cu ratio

The Zn: Cu ratio in each region was calculated (Fig 2) and found to average around 50 in both islet and peri-islet regions in non-diabetic tissue from young and old mice. As expected, this was lower in exocrine regions. Young Db mice had significantly decreased Zn: Cu ratio in islets (28.9 ± 2, *P *< 0.05) and peri-islet regions (28.3 ± 3 *P *< 0.05). This was abrogated in old Db mouse islets, although peri-islet regions still tended towards decreased Zn: Cu ratios. Overall, non-diabetic control islets had a Zn: Cu ratio 1.5-fold higher than Db islets (*P *< 0.01).

Immunofluorescence

### Islet hormones

**Figure 4:**
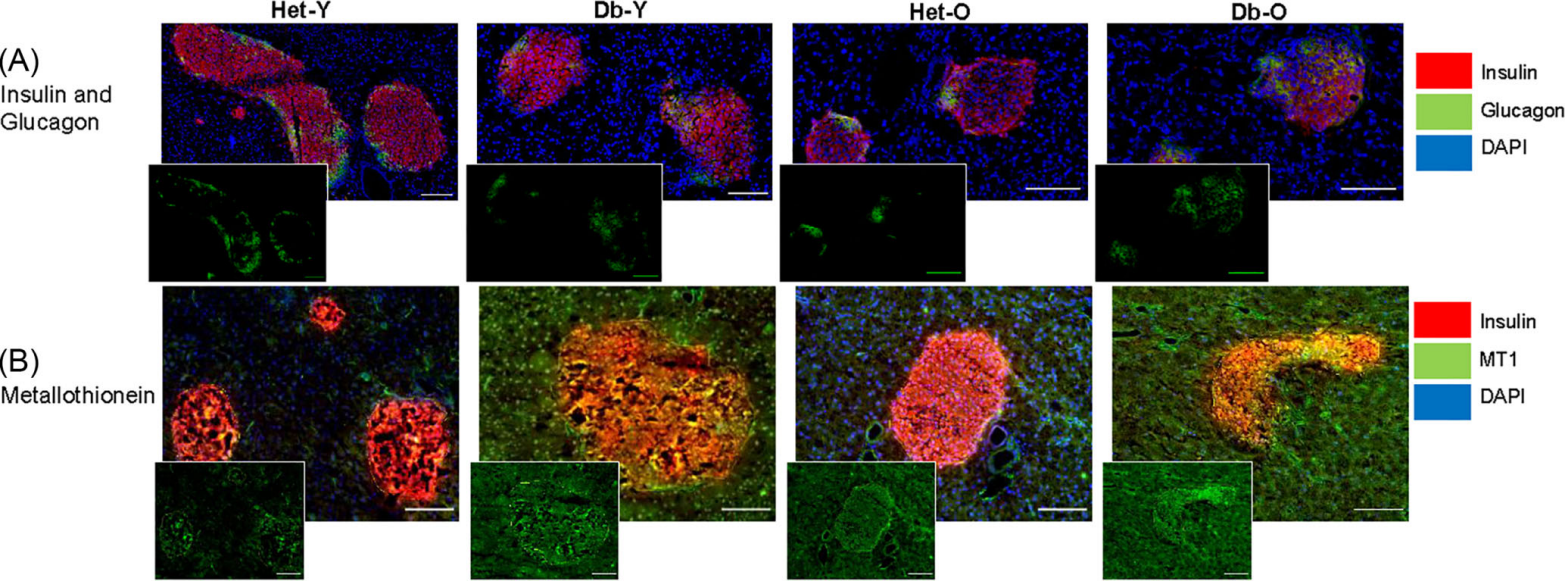
**Immunofluorescent microscopy of insulin, glucagon and metallothionein**. Adjacent sections were stained with anti-insulin (red), DAPI (blue) and either anti-glucagon or anti-metallothionein (green, panels (**A**) and (**B**), respectively). One representative sample from each group is depicted here. Islets stained more intensely than exocrine tissue for metallothionein in the diabetic and old mice. Staining brightness is relative within panels. Scale bar = 100 μm.

**Figure 5:**
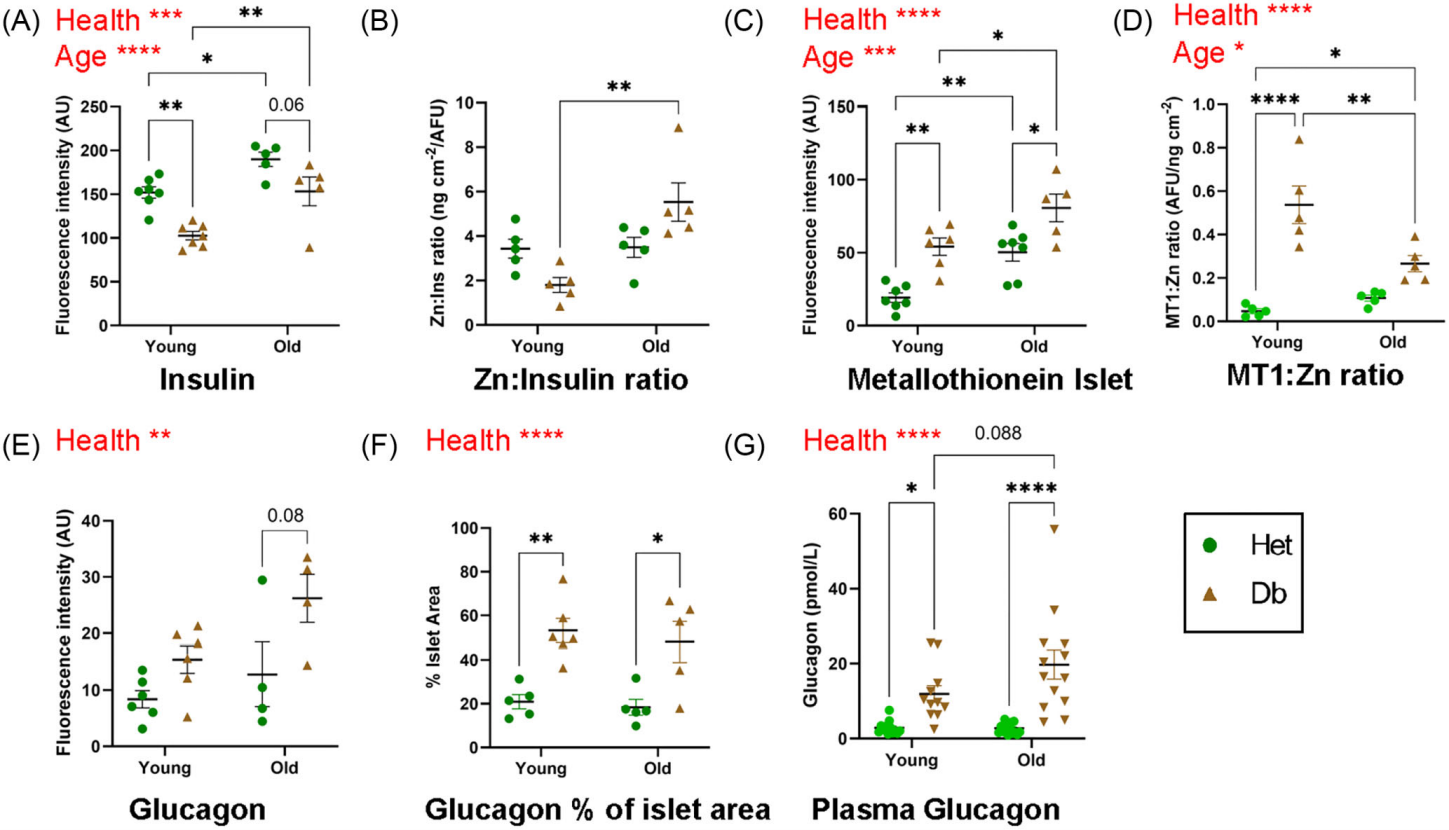
**Insulin, glucagon and metallothionein quantitation within islets**. Fluorescence intensity was quantified in ImageJ and data is presented for Het (green circles) and Db (tan triangles), young and old groups. Where a statistically significant difference was found for Het vs Db groups collectively, it is indicated by the word 'Health' above the figure: similarly for 'Age'. Each data point represents the average staining intensity of all islets in one pancreas section per animal (mean 10 islets per animal). (**A**) Fluorescence intensity of insulin was significantly higher in healthy islets, and also increased with age. (**B**) The ratio between Zn and insulin fluorescence remained constant between young and old healthy mice. However, the old diabetic group demonstrated a significantly higher Zn:insulin ratio. (**C**) Metallothionein fluorescence intensity in the islet region increased both in aging and insulin resistance/diabetes. (**D**) MT1: Zn ratio increased in diabetes. (**E**) Glucagon fluorescence intensity of the islet region was increased in diabetic animals, though this did not reach significance between individual groups. (**F**) The distribution of glucagon across the islet area was increased in diabetic animals of both ages. (**G**) Plasma glucagon measured by ELISA supported the increase in glucagon in diabetic animals measured by immunohistochemistry. Het: control mice (heterozygous), green circles; Db: obese, diabetic (db/db mouse model for type 2 diabetes), tan triangles. *P < 0.5, **P < 0.01, ***P < 0.001, ****P < 0.0001.

**Figure 6:**
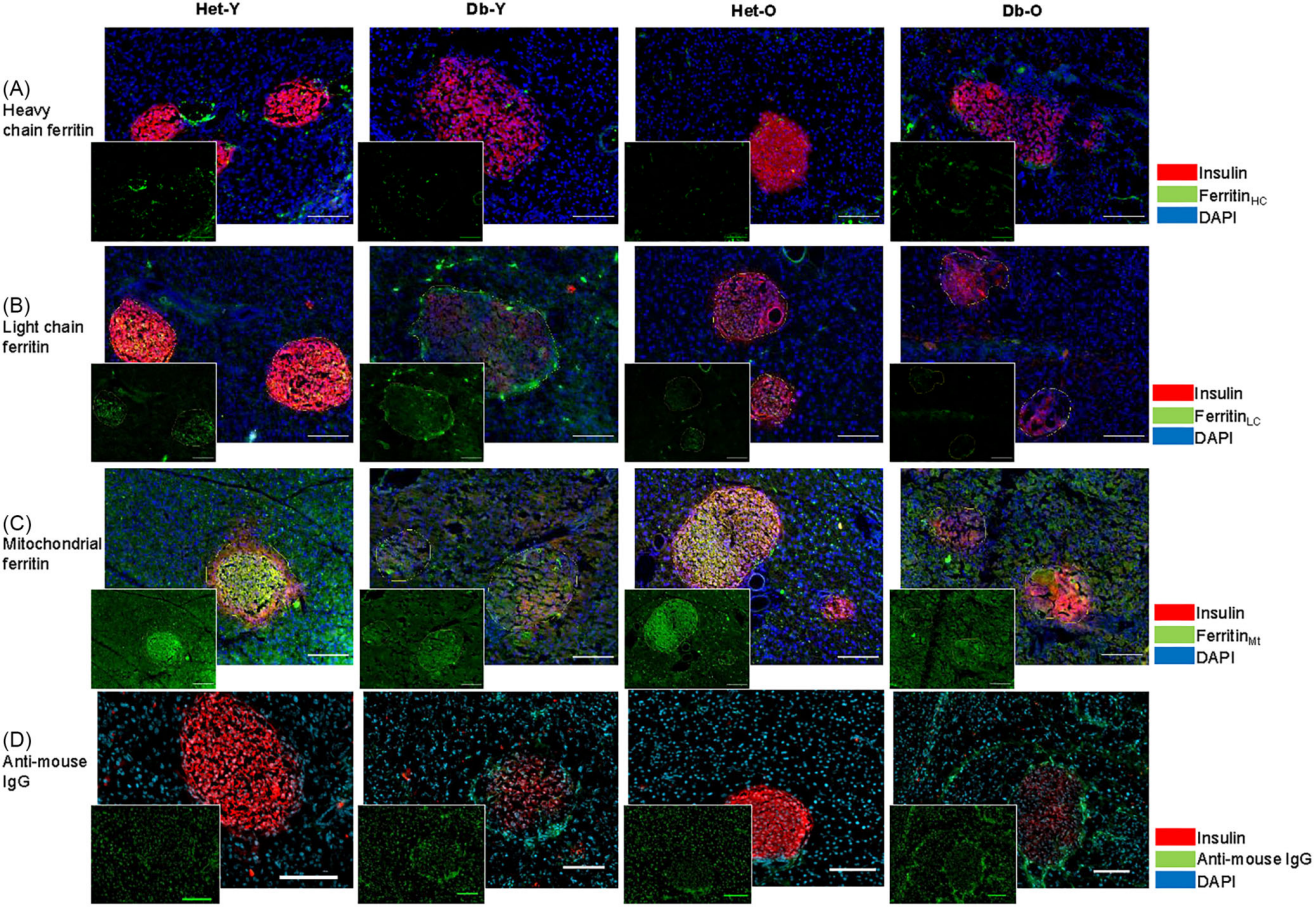
**Immunofluorescent microscopy of insulin, ferritins and IgG**. Adjacent sections were stained with anti-insulin (red), DAPI (blue) and either anti-heavy chain ferritin, anti-light chain ferritin or anti-mitochondrial ferritin (green, panels **A, B** and **C**, respectively). One representative sample from each group is depicted here. Islets stained more intensely than exocrine tissue for light chain and mitochondrial ferritin. Staining brightness is relative within panels. Scale bars = 100 μm.

**Figure 7:**
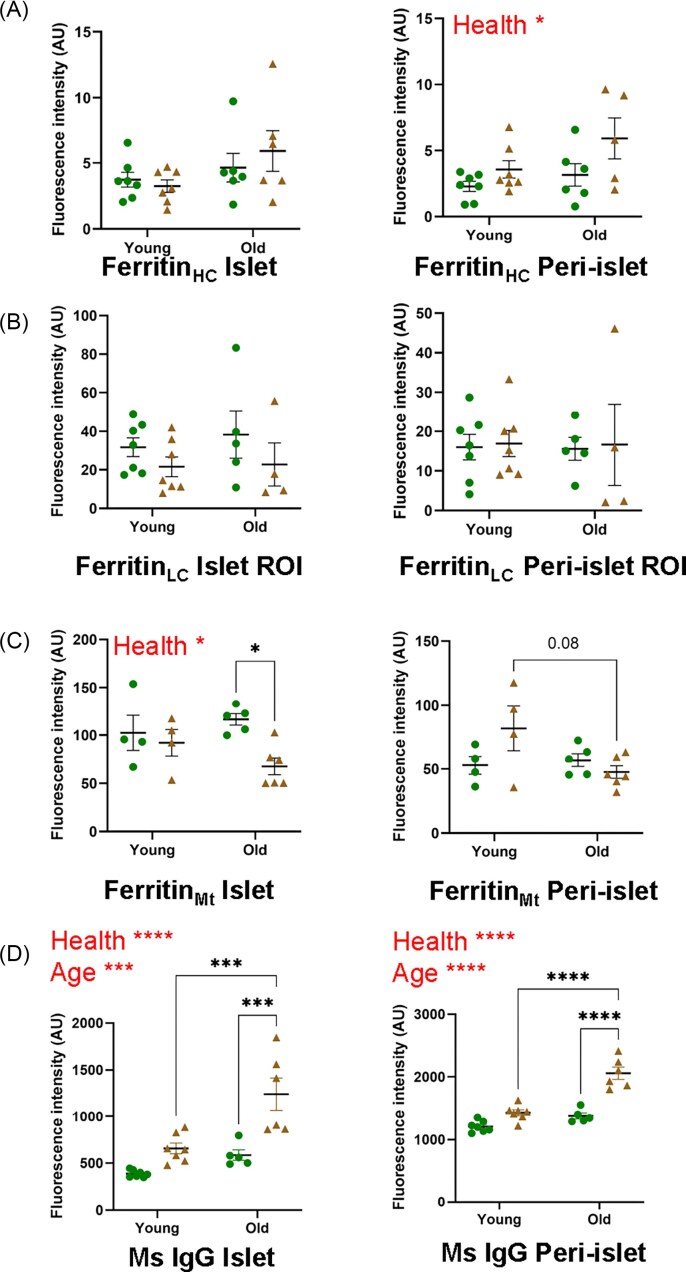
**Ferritins and IgG quantitation**. Fluorescence intensity was quantified in ImageJ or ZEN 3.8 (mouse IgG data) and data is presented for control (Het, green circles) and diabetic (Db, tan triangles), young and old groups. Where a statistically significant difference was found for Het vs Db groups overall, it is indicated by the word ‘Health’ within the figure. Similarly, significant differences between young and old groups are denoted by the word ‘Age'. The left- and right-hand panels present data for the islet and peri-islet regions of interest (ROIs), respectively. Each data point represents the average of all islets in one pancreas section from a unique animal (mean 10 islets per animal). Panel (**A**) gives mean fluorescence data for heavy chain ferritin; panel (**B**) for light chain ferritin; panel (**C**) for mitochondrial ferritin; and panel (**D**) for anti-mouse IgG (used as a proxy for inflammation). *P < 0.5, **P < 0.01, ***P < 0.001, ****P < 0.0001.

To further investigate elemental changes in islets, immunofluorescent staining was undertaken against functional and metal-binding proteins of interest (Fig 4–7). Given that beta cells are known to have higher Zn content than other pancreatic islet cells, and that alpha and beta cells are the two most prolific cell types in islets, immunostaining against insulin, representing beta cells, and glucagon, representing alpha cells, was undertaken. There was significantly decreased fluorescence intensity of insulin staining in the diabetic vs the non-diabetic islets, as expected (*P *< 0.0001, young and old) (Fig 4A, 5A). Zn: insulin ratio was significantly higher in age (*P *< 0.01) and in old compared to young diabetic animals (*P *< 0.001, Fig 5B).

Fluorescence intensity of glucagon staining was not significantly different between young and old, or diabetic and non-diabetic islets. However, glucagon immunoreactivity was distributed over a broader area of the diabetic islets in both age-groups (*P *= 0.0053, young; *P *= 0.014, old, Fig 4A, 5E, F). Plasma glucagon measured by ELISA was also significantly increased in diabetic animals in both age groups (4-fold, *P *< 0.05 and 7-fold, *P *< 0.0001 in young and old mice, respectively, Fig 5F). Db islets appeared to be more heterogeneous in Zn fluorescence intensity than Het islets, possibly due to the increased α-cell regions.

### MT1

Metallothionein 1 (MT1) was measured using immunofluorescent methods to determine whether changes in MT1 correlated with Zn abundance and/or speciation patterns observed. In islet regions, MT1 was upregulated in an age- (*P *= 0.005, Het; *P *= 0.04, Db) and diabetes-dependent manner (*P *= 0.0024, young; *P *= 0.0126, old) in murine islets (Fig 4B and 5C). MT1: Zn was calculated (Fig 5D) and Db islets were found to have a significantly elevated ratio (*P *< 0.0001).

### Ferritins

To assess whether changes in Fe abundance in the peri-islet and exocrine regions were due to increased storage, we used antibodies against mitochondrial ferritin (Fer_Mt_), heavy chain ferritin (Fer_HC_) and light chain ferritin (Fer_LC_) (Fig 6,7). There was a small but significant decrease in mitochondrial ferritin in old diabetic compared to non-diabetic islets (*P *= 0.03). No other significant changes in immunoreactivity for any of the ferritins were observed in the islet or peri-islet regions. Distribution patterns were different for different ferritins. Fer_LC_ was localised within the islets while Fer_HC_ was largely found in exocrine regions. Fer_Mt_ was present in exocrine and endocrine regions although with higher expression in islets.

### Immunoglobulin G (IgG)

IgG is the most abundant antibody in blood and increases during inflammation. We used anti-mouse IgG as a proxy for inflammation and found that it was increased in islets and peri-islet regions in diabetes (*P *< 0.0001) and age (*P *< 0.001) (Fig 6 and 7D).

### X-ray Absorption Near-Edge Spectroscopy (XANES) imaging

**Figure 8:**
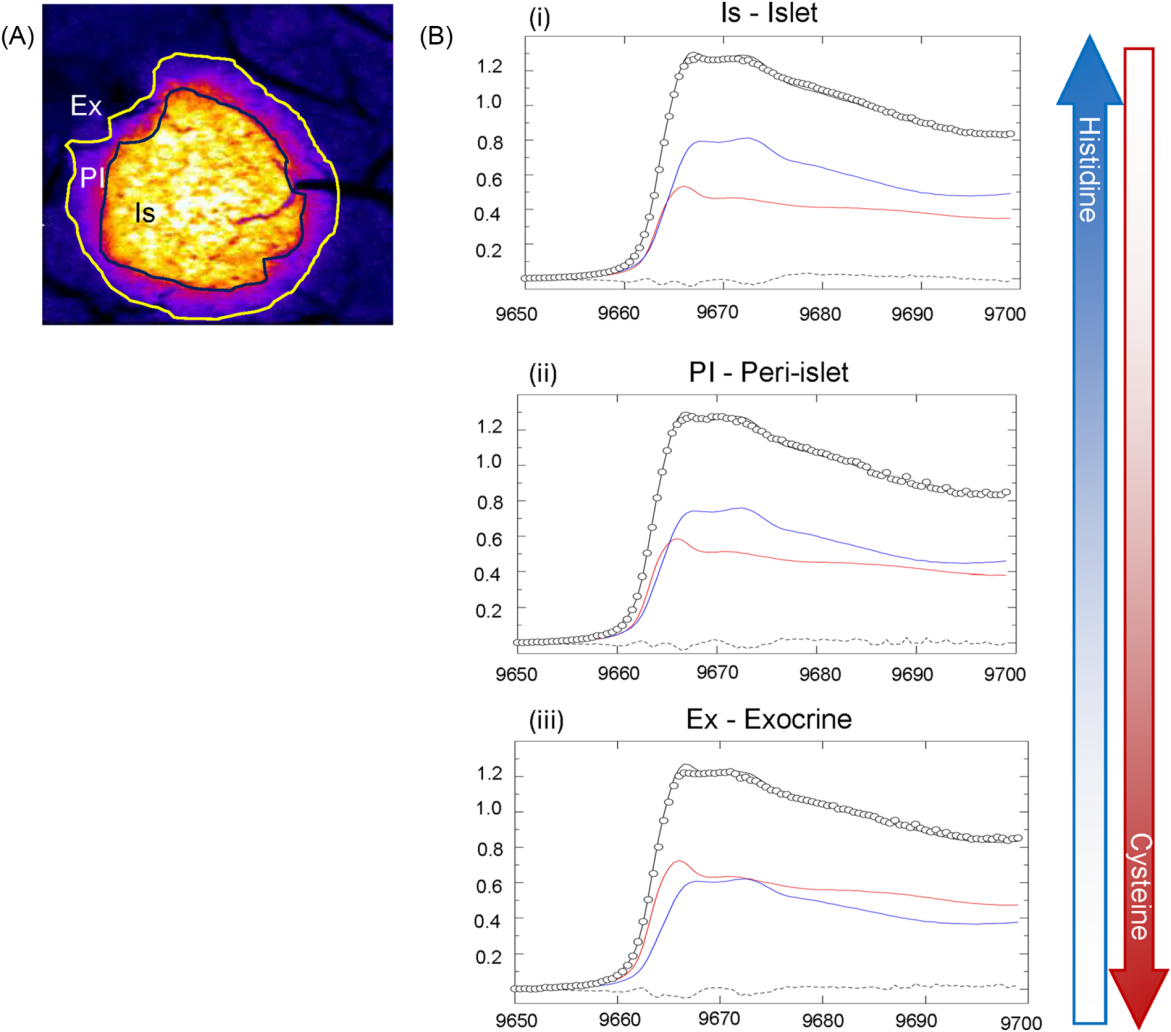
**Zn speciation was altered across anatomical regions**. Zn speciation was investigated in different anatomical regions of healthy pancreatic tissue (**A**) including islets (Is), the peri-islet region (PI) and exocrine regions (Ex). After fitting to a Zn speciation library previously published by our group (46), histidine and cysteine were determined to be the most abundant Zn ligands. Average speciation changed between anatomical regions, with histidine being the dominant ligand within islet areas (**B_i_**). Cysteine became more dominant with distance from the high Zn islet region (**B**_ii, iii_), overtaking histidine as the dominant species in exocrine tissue (**B**_iii_). Data for (**B**) represent averages from n = 5 samples, with error bars removed for clarity.

To see whether changes in Zn location and abundance were concomitant with changes in its speciation, we fitted Zn K-edge XANES spectra from islet, peri-islet and exocrine regions in non-diabetic tissue samples to a spectral library of biologically relevant compounds created in our laboratory [46]. Differences were found in speciation between high- and low-Zn content regions, representing islets and exocrine tissue, respectively (Fig 8). The best fit (smallest residuals) was achieved using spectra from standard solutions of Zn^2+^ + excess histidine [modelling coordination of Zn^2+^ to imidazole nitrogen of histidine residues, referred to as Zn(histidine) from now on] and Zn^2+^ + excess cysteine [modelling coordination of Zn^2+^ to thiol sulfur in cysteine residues, referred to as Zn(cysteine) from now on]. Interestingly, in non-diabetic Het animals the contribution of Zn(histidine) to the average spectra demonstrated a gradient that correlated positively with Zn^2+^ abundance, such that Zn(histidine) contributed more to spectra collected in the islet region but less in the exocrine region. Conversely, coordination of Zn^2+^ to cysteine exhibited an inverse correlation with Zn concentration, contributing less to spectra from the islet region and dominating in the exocrine region. Within islet regions, a noticeable difference was observed in Zn speciation between young Het mice and young *db*/*db* mice, the latter displaying increased Zn(cysteine) and decreased Zn(histidine). Interestingly, there was a counterintuitive reversal of this pattern in old diabetic islets, where a ratio of Zn(histidine): Zn(cysteine) was observed similar to that of wild type islet regions despite more advanced diabetes.

**Figure 9:**
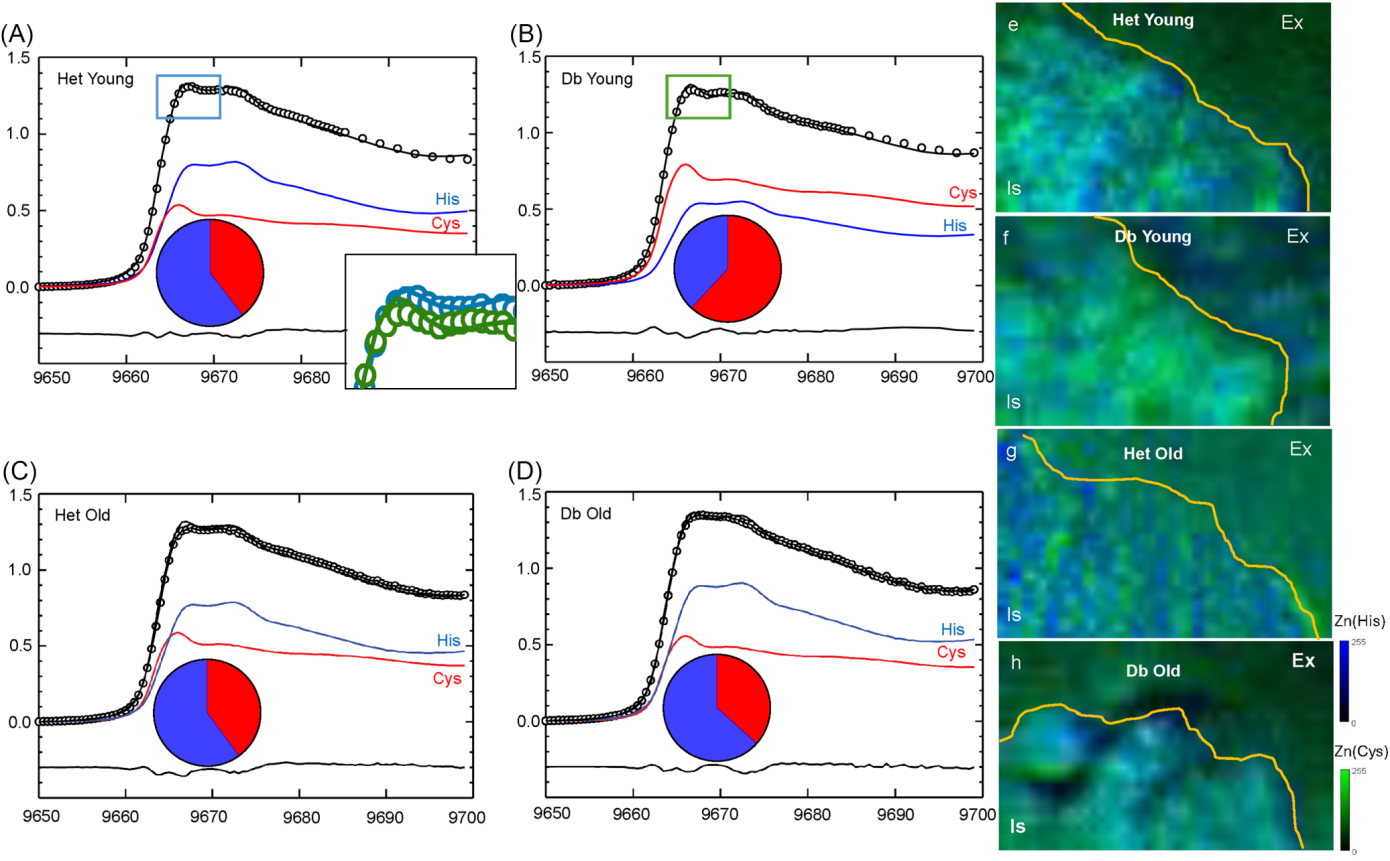
**Islet Zn speciation was altered between healthy and diabetic animals**. Zn speciation was investigated in islet regions across young (14 weeks, **A, B**) or old (28 weeks, **C, D**), healthy (**A, C**) and diabetic animals (**B, D**). Both control groups and old diabetic mice demonstrated similar speciation with a greater contribution from Zn(histidine) species, while young diabetic mice exhibited predominantly cysteine speciation, similar to exocrine regions in healthy animals. The inset in (**A**) shows an overlay of the peaks within the rectangles (Het young, blue and Db young, green). The fraction of fit for cysteine and histidine speciation within the islet region is given in the pie graphs. Each graph represents mean data from n = 5 animals per group. For clarity, error bars are not shown. A representative sample of each group is also displayed as a chemically specific, semi-quantitative speciation map of Zn coordinated to cysteine (green) and histidine (blue) (**E-H**). Ex = exocrine, Is = islet, calibration bars for Zn(histidine) (blue) and Zn(cysteine) (green) are in relative grey values.

Examination of the eigenvalue plot from principal component analysis (PCA) determined that there were likely to be between 2–4 significant spectral components per sample (Supplementary information Fig S2). Considering both PCA and linear combination fitting of average XANES spectra, where multiple fits to a variety of standard solutions and model compounds were trialled, it was determined that adequate fits with the lowest residuals could be obtained from fitting to spectra modelling Zn(histidine) and Zn(cysteine) coordination. Following PCA, single value decomposition (using standard solution spectra as spectra models) was performed to produce chemically specific Zn speciation maps of Zn coordinated to cysteine, and Zn coordinated to histidine (Fig 9, Fig S2).

## Discussion

Despite the well-known link between Zn and diabetes, few studies have used methods that allow the direct mapping of Zn across pancreatic tissue. A recent study successfully mapped Zn and other elements in isolated islets using XFM [75], but the current study using frozen pancreatic slices has the advantage of visualising islets in their tissue context without the considerable disruption of islet isolation. To our knowledge this is the first reporting of XFM-based elemental analysis and the first Zn micro-XANES imaging analysis in fresh frozen tissue across the exocrine and endocrine murine pancreas. This work demonstrates variations in concentrations of six elements (Cl, K, Ca, Fe, Cu and Zn) across three regions of the pancreas (islets, peri-islets and exocrine tissue), and how they are affected by ageing and type 2 diabetes. In addition, we demonstrate regional variations in Zn speciation across islets and exocrine regions, and how this aligns with the major Zn-binding proteins, insulin and MT1. Finally, we reveal how Zn speciation is affected by diabetes and ageing. This work increases our understanding of the relationship between Zn, diabetes and ageing.

### Post-XFM insulin staining confirmed that islet regions coincided with regions of Zn enrichment

During XFM imaging, islets were initially identified for higher resolution imaging by searching for circular regions of high Zn fluorescence in an initial low-resolution scan. To confirm that selected regions were indeed islets, we post-stained the same tissue sections with anti-insulin and anti-glucagon antibodies. Although X-ray damage from cryo-XFM with a dwell time of 2 ms mildly increased background fluorescence, the tissue was still compatible with immunohistochemical staining [76]. Areas used for XANES spectroscopic imaging, with an effective dwell time of 200 milliseconds, were not compatible with post-XANES immunofluorescent staining, most likely due to photodamage, such as denaturation, cross-linking or conformational changes associated with irradiation in the target proteins [77–80]. Regions of high Zn were found to co-localise with insulin staining, confirming islet location, despite some shrinking and cracking during air drying.

### Elemental mapping revealed changes in anions and cations that regulate secretion

Electrically charged ions and their regulated flux through specific ion channels are required to establish the membrane potentials utilised to initiate secretory mechanisms in excitable and non-excitable cells such as pancreatic beta and alpha cells and acinar cells, respectively [81–83]. Chloride (Cl^−^) and potassium (K^+^) are important ions that work synergistically to hyperpolarise and depolarise the plasma membrane during resting and secretory phases [84]. Calcium ions (Ca^2+^) are also key signalling ions responsible for granule exocytosis in many tissue types, including pancreatic endocrine and exocrine cells.

### Chloride

Recent literature addressing chloride anion physiology in islets is scarce. However, since 1978 it has been known that chloride flux influences beta cell electrical activity [85]. A high chloride concentration (∼34 mM, over 3-fold greater than thermodynamically predicted [84]), is maintained against its electrochemical equilibrium in beta cells, which effluxes during passive channel opening. This is due to a variety of specialised chloride transporters and channels gated by ATP, cell volume, the neuro-transmitters GABA and glycine, and Ca^2+^ in a complex system that is poorly understood and remains controversial, recently reviewed by Di Fulvio and Aguilar-Bryan [84]. Alpha cells, on the other hand, have lower chloride concentrations and constitutively export chloride ions due to the expression of potassium chloride co-transporter 2 (KCC2) channels in the cell membrane [86]. GABA co-released with insulin from beta cells binds to its receptor (GABA_A_) on alpha cell membranes which, being a chloride channel, stimulates chloride ion flux into the alpha cell, causing hyperpolarisation. This is thought to be a mechanism that causes disparate responses to GABA stimulation in alpha and beta cells, being inhibitory in the former and excitatory in the latter [86]. In diabetic beta cells, Cl^−^ regulation and thus membrane potential is altered [87,88]. We report elevated chloride concentrations in Db islets, which is consistent with the increased accumulation of labelled Cl^−^ and higher estimated Cl^−^ concentrations in Db compared to healthy islets reported by Berglund and Sehlin [89]. Dysregulated chloride flux may help explain poor glucose coupling and increased basal, non-stimulated insulin release in these animals [87].

In exocrine regions, chloride was measured at higher concentrations than in endocrine regions in healthy (*P *< 0.001) but not diabetic mice. Cl^−^ channels are important in exocrine fluid secretion [83] and the dysfunctional chloride channel in cystic fibrosis, cystic fibrosis transmembrane conductance regulator (CFTR), is the most researched chloride channel in the exocrine pancreas.

### Potassium

K is well-known to be the predominant intracellular cation in mammalian cells and plays a major role in maintaining the electrical potential in cell membranes. Glucose-stimulated insulin secretion relies on K^+^ flux, since ATP-dependent K channels (K_ATP_) in the beta cell membrane respond to changes in the ATP: ADP ratio after glucose metabolism to initiate plasma membrane depolarisation and insulin secretion [90–92]. While it is difficult to speculate on physiological consequences of increased K presence in Db islets without further knowledge of K_ATP_ activity, we do know that K dysregulation is associated with diabetes. This is partly due to the influence of insulin to stimulate K uptake in insulin-sensitive tissue via the Na^+^-K^+^-ATPase ion pump [93,94]. In insulin deficiency, reduced tissue uptake can result in higher plasma K levels [95,96]. *Db*/*db* mice are hyperinsulinaemic [60], likely contributing to intracellular K uptake [97].

### Calcium

Calcium signalling is required for secretion of zymogens from exocrine tissue and hormones from endocrine tissue and dyshomeostasis can cause disease, thus it is tightly controlled across the pancreas [98–100]. It is not immediately clear why Ca accumulated in the peri-islet region, or whether this accurately reflects the *in vivo* situation. It is worth mentioning that these animals were not fasted before death, and the prandial state of the individual is known to influence Ca^2+^ flux in both endocrine [101] and exocrine [102] tissue. To our knowledge, this is the first time calcium has been mapped across both exocrine and endocrine pancreas, demonstrating calcium accumulation in the peri-islet region. Further research to discover whether this could act as a calcium storage zone or has other mechanistic implications would be of interest.

### Elemental mapping with correlative immunofluorescence imaging reveals heterogeneous distribution and diabetes-influenced changes in essential biometals

Three essential transition metals, all of which have been linked to the pathogenesis of diabetes [103], were mapped in this work: Fe, Cu and Zn. Each of these, among other functions, are required for transient roles such as for enzyme catalysis and therefore must be available in strictly controlled, low concentrations as ‘free’ or loosely bound pools within cells [104], also referred to as ‘labile’ pools. Each has been associated with a variety of pathologies when concentrations are either elevated or diminished [104]. These elements may also influence each other, for example, by competing for binding sites in metallothioneins. Dietary zinc competes with copper for uptake, and therefore excessive zinc supplementation can manifest as copper deficiency [58,105]. Extended periods of excessive zinc ingestion can also result in depleted iron stores [106]. Both Cu and Zn were depleted in diabetic islets, while Fe was elevated, not in the islets, but in the peri-islet and exocrine regions of diabetic tissue.

### Changes in Fe were not associated with increased ferritin for Fe storage

Fe dysregulation has previously been linked to diabetes [107], however, Fe accumulation did not occur in islet regions in the current study. The Fe elevation in diabetic peri-islet and exocrine regions could be related to inflammation. Our IgG studies suggest a significant increase in inflammation in all regions including islets, with strong fluorescence particularly noticeable around blood vessels in peri-islet regions (Fig 6D).

Heavy chain, light chain and mitochondrial ferritin immunofluorescence results suggest that Fe storage was not up-regulated in Db islet or peri-islet regions. Rather, old Db mice had a small decrease in mitochondrial ferritin in their islets. Each ferritin molecule has the capacity to store up to 4500 Fe ions [108], allowing for considerable variation in Fe content for a given number of ferritin proteins. Interestingly, the gene for Fer_LC_ has recently been recognised as beta-cell-specific within islets, since it is not associated with other pancreatic endocrine cell types [109]. Despite the anticipated lower number of beta-cells in diabetic islets, only a small, non-significant depletion of light chain ferritin was observed in diabetic islets.

### Copper

Copper is an essential cofactor in several enzymes involved in redox reactions and which are employed in roles as diverse as signalling, oxidative phosphorylation, iron metabolism and melanin synthesis, among others [104]. Dietary copper deficiency selectively causes acinar cell apoptosis, leaving islet tissue unaffected [110,111]. We found that copper concentrations in islets were higher than exocrine regions in non-diabetic control tissue. While there was loss of copper from islets but not from exocrine regions in diabetes, copper concentrations were still somewhat elevated in islets compared to exocrine regions.

### Zinc

Two important pools of labile Zn in islets are a) Zn stored in insulin granules, where insulin hexamers crystalise around two Zn^2+^ ions [112,113], interacting with the N and O in histidine side chains [48,114,115], and b) cytoplasmic Zn transiently bound to Metallothionein 1 (MT1) where seven Zn^2+^ ions bind with tetrahedral coordination to sulfur in cysteine residues [116–120]. The predominance of these Zn pools in islet biology leads to expectations that their effects on the labile Zn pool are detectable. Our data supports this for groups other than the old diabetic mice.

### Zn and insulin correlated in groups other than old diabetics

The large relative size of the insulin-bound Zn pool suggests that Zn and insulin content could be expected to correlate, since insulin is stored and secreted in 3:1 stoichiometry with Zn. We demonstrate a significant correlation between Zn and insulin in these groups (Pearson’s r = 0.76, r^2^ = 0.57, p = 0.001, Db-O group excluded). However, the Zn: insulin ratio in the Db-O group was higher than expected (Fig 5B) due to relative insulin depletion while Zn content remained similar to non-diabetic islets.

High insulin (and hence Zn) secretion rates, as evidenced by high plasma insulin measurements (Supplementary Fig. S1A), are likely responsible for decreased insulin and Zn concentrations in young diabetic islets as found in ours and other studies [121,122]. However, decreased insulin still correlated with Zn abundance in these animals. In older diabetic animals, plasma insulin was only moderately elevated compared to non-diabetic control animals despite the high blood glucose (Supplementary Fig. S1B), possibly due to beta-cell fatigue and loss.

In agreement with the increase in glucagon secretion in diabetes found in ours (Fig 5F) and other studies [123–125], glucagon immunofluorescent staining was consistent with a relative increase in the number of alpha cells in the islets [126]. It is known that beta cells can dedifferentiate or transdifferentiate into a more alpha cell phenotype in hyperglycaemic environments [127–129] and this could partially explain a reduction in Zn content in diabetic islets, since alpha cells are less zinc-rich than beta cells [130].

### Metallothioneins

Metallothioneins, which regulate intracellular Zn^2+^ signalling and availability by buffering or muffling cytosolic Zn^2+^, act as a Zn re-distributor and transient reservoir [131,132]. Since the ionisation state of the thiol group determines its affinity for Zn^2+^ [133], and its pK_a_ is close to physiological pH at 7.4 [134], it has the capacity for sensitive responses to small changes in the immediate environment [135]. This makes it both exquisitely aligned for signalling roles but also susceptible to dysfunction in altered environments such as in oxidative stress.

MT1: Zn ratios provide limited information and don’t necessarily reflect metal content or binding efficacy which is affected by the redox state and presence of biological compounds such as ATP and glutathione [136]. However, the increase in MT1: Zn ratio in diabetes and between young and old diabetics is, at the least, evidence of disrupted Zn regulation.

Furthermore, the affinity of copper for metallothionein is higher than that of zinc [132] and can thus displace zinc from metallothioneins, with implications for zinc bioavailability. Decreased copper concentrations such as were found in diabetic tissue would therefore be favourable in zinc-diminished islets. However, Zn: Cu ratios were still lower in diabetic tissue, particularly in islet and peri-islet regions in young mice.

Interestingly, emerging evidence suggests that MT1 has a diabetogenic effect in some contexts [116,137]. MT1 upregulation (such as in our results) is associated with beta cell failure and T2DM while downregulation is observed during beta cell compensation. It remains unclear whether Zn, the primary MT1 ligand in islets, and its speciation (when not bound to MT1) and perhaps also its competition with Cu^2+^, may have a role in these complexities.

### *Zn speciation correlated with insulin and MT1 abundance* in *groups other than old diabetics*

In islets from young mice in our study, the depletion of insulin and increase in MT1 in Db compared to non-diabetic control samples is reflected in the lower contribution of Zn(histidine) (representing insulin) and higher contribution of Zn(cysteine) (representing MT1) binding to Zn within the islet ROI in the former group. Further, old non-diabetic groups presented similar alignment of speciation with relative insulin and MT1 content. This provides evidence that insulin and MT1 binding influence average Zn speciation in islets. The relative changes in insulin and MT1 abundance align with Zn abundance and coordination found in islet vs exocrine regions and non-diabetic vs diabetic mice in all but the old diabetic cohort.

Both the unexpectedly high Zn: insulin ratio and the persistence of high Zn(histidine) binding in the old Db group islets, concomitant with low insulin and high MT1 abundance, suggests Zn dyshomeostasis in this group. We hypothesise that further non-insulin Zn(histidine) complexes are present in advanced diabetes and speculate that this altered speciation is potentially diabetogenic.

Putative mechanisms for the higher Zn concentration and Zn(histidine) speciation in old diabetic islets include a) restoration of Zn homeostasis and speciation by compensatory mechanisms; b) altered binding affinity of MT1 for Zn [138] driven by hyperglycaemia-related oxidative stress [139] and resulting in elevated disordered ‘free Zn’ concentrations in intracellular regions; or c) a combination of a) and b) where compensatory mechanisms such as upregulation of Zn transporters compound the excess of ‘free Zn’. In case a), Zn supplementation would have limited effects on the treatment of T2DM as is reported elsewhere [42]. In b) and especially c), signalling by ‘free Zn’ would be expected to upregulate MT1 expression (as seen in our results) via metal responsive element transcription factor-1 [116]. Since Zn muffling requires that MT1 and Zn transporters work in concert to shunt Zn to specific compartments such as insulin granules [20,132], failure of MT1 to deliver Zn for specific cellular requirements may cause localised Zn insufficiency even in a context of excess ‘free Zn’ (bound to available histidine residues), potentially driving a ‘vicious cycle’ of Zn dyshomeostasis. Increased background ‘noise’ from displaced Zn and its diluting effect on the small concentration changes required for Zn^2+^ signalling could have a significant negative impact on islet function. Further study is required to clarify relevant mechanisms.

### Study limitations and future directions

A limitation of this study relates to the selection of regions of interest for XFM and XANES imaging. Since it is well known that high Zn concentrations are found in pancreatic islets, and since any form of pre-staining would alter labile Zn, regions of interest were selected visually according to areas of high Zn found in the tissue. This could introduce bias in the results such that the islets with highest Zn content were selected. However, the nature of the bias would likely reduce rather than enhance differences found between groups, since higher Zn islets were more difficult to find in diabetic animals, possibly reflecting more variation in Zn content. Post-XFM staining with antibodies directed against insulin and glucagon was used to minimise potential bias and to confirm that regions of high Zn were indeed islets.

Due to time and access limitations of the synchrotron X-ray methods used in this study, just one or two islets per pancreas were measured. This is a very limited sample size in a tissue type known to display high variation, and further study would be beneficial.

Future directions include a study of the relationship between oxidative stress and Zn abundance in diabetes. Zinc supplementation studies in this diabetes model would also be beneficial.

### Summary

This study reports age- and diabetes-specific changes recorded by XFM in biologically relevant elements including three ions important in secretion mechanisms (Cl, K, Ca) and three transition metal ions known to be associated with diabetes (Fe, Cu, and, most significantly, Zn). Correlative immunofluorescence studies determined that depletion of insulin and increased distribution of glucagon was associated with significantly depleted Zn in young diabetic islets. Zn content was normalised in islets from old diabetic mice, despite higher disease burden and persisting low insulin content along with widespread glucagon distribution. It is not known whether the increased Zn-to-insulin ratio in old diabetic mice contributes to metabolic impairment. Zn speciation changes were associated with Zn, insulin and MT1 abundance, with more histidine speciation in high Zn regions and more cysteine speciation correlating with low Zn regions. This related to different tissue types (endocrine vs exocrine regions) and where Zn and insulin depletion together with MT1 upregulation was associated with early diabetes. Zn speciation became dysregulated in advanced diabetes, characterised by higher-than-expected histidine speciation in the absence of increased insulin and presence of increased MT1. A putative mechanism for these changes involving a vicious cycle of oxidative stress affecting MT1-Zn binding and subsequent Zn redistribution is given. These findings support the growing body of evidence around the association of Zn regulation and diabetes and demonstrate the value of emerging correlative imaging techniques in this field of research.

## Supplementary Material

mfaf043_Supplemental_File

## Data Availability

Data is available upon reasonable request to the authors.
