# Supplementary material for: Correlative imaging reveals metal dyshomeostasis and altered zinc coordination environments in a pre-clinical Type 2 diabetes model
Source: Metallomics. 2026 Jan 2;18(1):mfaf043. doi: 10.1093/mtomcs/mfaf043 (PMC12859259; doi:10.1093/mtomcs/mfaf043)
Supplement: mfaf043_Supplemental_File [file mfaf043_supplemental_file.pdf]

Supplementary data.

Correlative imaging reveals metal dyshomeostasis and altered zinc coordination environments in a pre-clinical Type 2 diabetes model

Gaewyn Ellison[1,2], Arazu Sharif[2,3], Meg Willans[1,2], Ashley Hollings[1,2], Ryu Takechi[2,3], Keith Bambery[4], Valerie Mitchell[4], Daryl L. Howard[4], Mark Hackett[1,2]*

[1]School of Molecular and Life Sciences, Faculty of Science and Engineering, Curtin University, Bentley, WA, Australia.

[2]Curtin Medical Research Institute, Curtin University, Bentley, WA, Australia

[3]School of Population Health, Faculty of Health Sciences, Curtin University, Bentley, WA, Australia

[4] Australian Synchrotron, ANSTO, 800 Blackburn Road, Clayton; VIC, 3168, Australia

**Corresponding author:* gaewyn.ellison@curtin.edu.au

Table S1. Model compounds included in the spectral library to which spectra from the tissue samples was fitted. The source of Zn^{2+} was $Zn(NO_3)_2$ dissolved in H_2O. Standard solutions were prepared with each ligand in ~3-fold excess. Each spectra therefore represents the average of all forms of Zn that occur in the presence of these ligands, as previously published. (See Hollings (46)).

Ligand	Coordinating Atom	Geometry
Chloride	Chlorine	Octahedral, tetrahedral
Phosphate	Oxygen	Uncertain
Glutamate	Oxygen	Distorted octahedral
Citrate	Oxygen	Octahedral
Water (pH 7)	Oxygen	Octahedral
Water (pH 13)	Oxygen	Tetrahedral
Histidine	Nitrogen	Tetrahedral
Cysteine	Sulfur	Tetrahedral

Table S2. Antibodies used in immunohistochemistry

Target	Abbreviation	Citation	Concentration	Conjugation
Insulin	Ins	(Santa Cruz Biotechnology Cat# sc-8033, RRID:AB_627285)	1 in 200	Alexa Fluor 564
Glucagon	Gcg	(Cell Signaling Technology Cat# 2760, RRID:AB_659831)	1 in 150	-
Ferritin (Heavy chain)	Fer_{HC}	(Abcam Cat# ab183781, RRID:AB_2940987)	1 in 400	-
Ferritin (Light chain)	Fer_{LC}	(Abcam Cat# ab69090, RRID:AB_1523609)	1 in 1000	-
Ferritin (mitochondrial)	Fer_{Mt}	(Abcam Cat# ab66111, RRID:AB_3665352)	1 in 100	-
Metallothionein 1	MT1	(Bioss Cat# bs-1328R, RRID:AB_10855955)	1 in 100	-
Rabbit IgG (secondary)	-	(Abcam Cat# ab150061, RRID:AB_2571722)	1 in 1000	Alexa Fluor 488
Mouse IgG	-	(Abcam Cat# ab150115, RRID:AB_2687948)	1 in 1000	Alexa Fluor 647

Table S3. Elemental quantitation in endocrine and exocrine tissue from healthy or obese, young or aged murine pancreas. All data is expressed as ng/cm^2, mean ± SEM. n = 5 animals per group.

[a] $p < 0.05$ compared to Het-Y controls. [b] $p < 0.05$ compared to Db-Y.
[c] $p < 0.05$ compared to Het-O

**Islet region**				
**Element**	**Het-Y**	**Db-Y**	**Het-O**	**Db-O**
Cl	1642 ± 186[b]	2653 ± 84[ac]	1333 ± 279[b]	2102 ± 133[c]
K	5434 ± 313[b]	8465 ± 538[a]	5088 ± 230[b]	7832 ± 240[ac]
Ca	194 ± 59	200 ± 67	175 ± 21	124 ± 15
Fe	73 ± 9	115 ± 21	112 ± 8	110 ± 12
Cu	7.6 ± 1.1	4.1 ± 0.4	9.1 ± 1.8[b]	7.6 ± 0.3
Zn	383 ± 46[b]	118 ± 16[a]	433 ± 40[b]	310 ± 24[b]
**Peri-Islet region**				
**Element**	**Het-Y**	**Db-Y**	**Het-O**	**Db-O**
Cl	2266 ± 157	2474 ± 88	2166 ± 132	2342 ± 100
K	6711 ± 266[b]	8100 ± 405[ac]	6706 ± 345[b]	8281 ± 166[ac]
Ca	271 ± 35	210 ± 43	324 ± 40	223 ± 15
Fe	66 ± 10	121 ± 29	90 ± 4	170 ± 31[a]
Cu	3.4 ± 0.5	2.5 ± 0.3	3.5 ± 0.6	4.5 ± 0.6
Zn	158 ± 13[b]	71 ± 12[a]	168 ± 14[b]	130 ± 13[b]
**Exocrine region**				
**Element**	**Het-Y**	**Db-Y**	**Het-O**	**Db-O**
Cl	2349 ± 162	2297 ± 93	2227 ± 79	2369 ± 117
K	7018 ± 298	7496 ± 294	6786 ± 441	8325 ± 208[ac]
Ca	200 ± 15	199 ± 25	255 ± 28	249 ± 14
Fe	67 ± 9	112 ± 17	91 ± 5	120 ± 13[a]
Cu	1.9 ± 0.4	1.8 ± 0.2	2.1 ± 0.2	2.3 ± 0.2
Zn	58 ± 2	50 ± 5	66 ± 3	65 ± 5

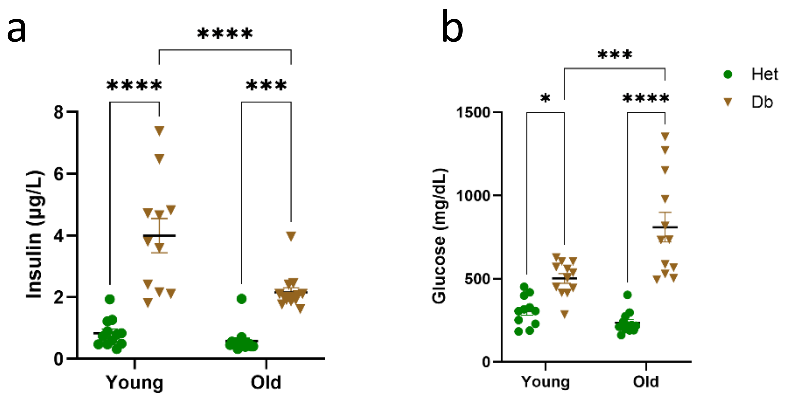

Figure S1: Non-fasting blood Insulin and glucose. (a) Non-fasting blood insulin measurements reveal elevated circulating insulin, particularly in young diabetics. Older animals with advanced diabetes show elevation, but not to the same degree, potentially due to beta cell exhaustion. (b) Despite the expected insulin resistance and response to the very high insulin levels, non-fasting blood glucose is elevated in young diabetics. In older diabetics with less circulating insulin, blood glucose is extremely high.

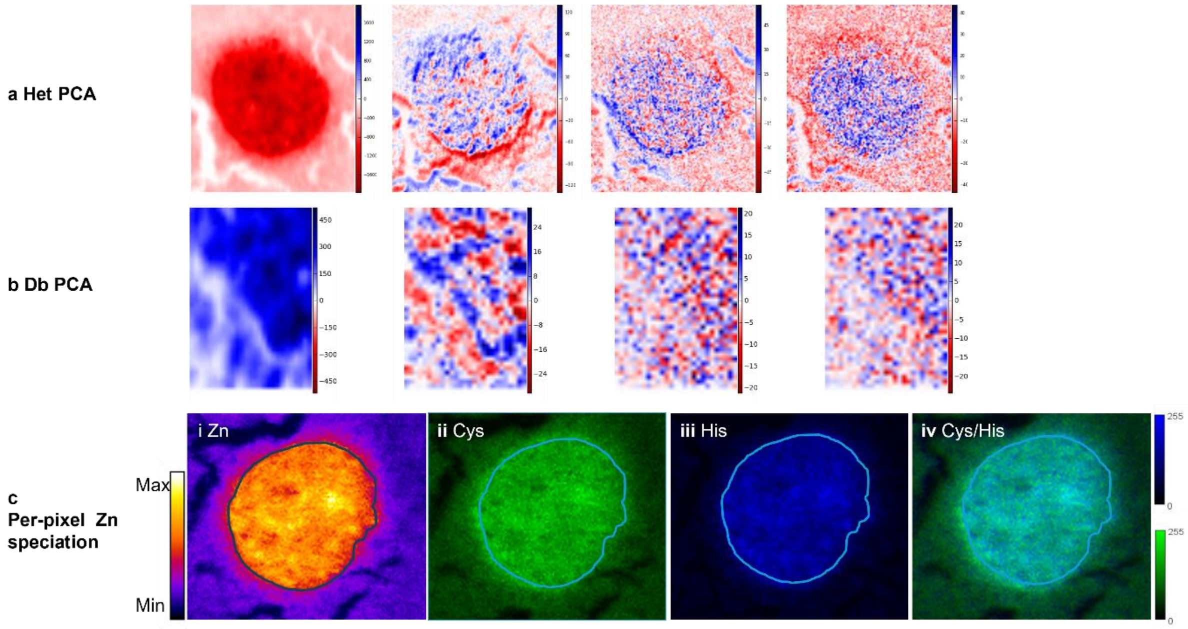

Figure S2: Principal Component Analysis (PCA) and Per-pixel speciation mapping. PCA was carried out in the young Het (a) and Db (b) groups. Eigenimages of the first four components from a representative young Het and Db sample (respectively) are shown, with an arbitrary intensity scale where negative values are shown in red and positive values in blue. Following PCA, the XFM Zn maps (c-i) were analysed by single value decomposition using standard solution spectra as spectra models to produce chemically specific Zn speciation maps of Zn coordinated to cysteine (c-ii, green) and histidine (c-iii, blue). An overlay of these two maps provided a semi-quantitative, chemically specific speciation map (c-iv).
